# Attenuated Total Reflectance-Fourier Transform Infrared (ATR-FTIR) Spectroscopy Coupled with Principal Component Analysis and Polymerase Chain Reaction (PCR) Assay for the Detection of Porcine and Bovine Gelatins in Dental Materials

**DOI:** 10.21315/tlsr2022.33.2.7

**Published:** 2022-07-15

**Authors:** Nining Irfanita, Widya Lestari, Mohamed Elwathig Saeed Mirghani, Irwandi Jaswir, Fitri Octavianti, Muhammad Salahuddin Haris

**Affiliations:** 1 International Institute for Halal Research and Training (INHART), International Islamic University Malaysia, 50738 Kuala Lumpur, Malaysia; 2 Department of Oral Biology, Kulliyyah of Dentistry, International Islamic University Malaysia, 25200 Kuantan, Pahang, Malaysia; 3 Avicenna Dental Clinic, Taman Danau Kota, 53300 Setapak, Kuala Lumpur, Malaysia; 4 Department of Pharmaceutical Technology, Kulliyyah of Pharmacy, International Islamic University Malaysia, 25200 Kuantan, Pahang, Malaysia

**Keywords:** Dental Material, Bovine Gelatin, Porcine Gelatin, ATR-FTIR, PCR, Bahan Pergigian, Gelatin Lembu, Gelatin Babi, ATR-FTIR, PCR

## Abstract

Muslims are prohibited from consuming products that contain pig products and their derivatives, including porcine gelatin. Medical and dental products are not exempt from the use of gelatin in their formulation. This study employs attenuated total reflectance-Fourier transform infrared spectroscopy (ATR-FTIR) coupled with principal component analysis (PCA) to detect and distinguish between porcine and bovine gelatins in dental materials. The results were further verified by polymerase chain reaction (PCR) assay. Species-specific primers targeting the 212 bp porcine cytochrome *b* and 271 bp bovine cytochrome *b* genes were used to amplify DNA in nine dental material samples. Detection and distinction of gelatin standards (bovine and porcine) against gelatin present in the dental materials was achieved using ATR-FTIR combined with PCA within wavenumber 1756 cm^−1^–1584 cm^−1^ (Amide I and Amide II). The detection limit for DNA was 0.001 ng/μL and 0.0001 ng/μL for bovine and porcine gelatins, respectively. Using PCR, one sample, BDM 01, was found to contain both porcine and bovine DNA, while one sample (BDM 14) was found to be positive for bovine DNA. The findings suggest that ATR-FTIR combined with PCA and conventional PCR are applicable for the identification of porcine and bovine gelatin in dental materials.

## Abstract (ms)

Umat Islam dilarang menggunakan produk yang mengandungi babi dan turunannya, termasuk gelatin babi. Produk perubatan dan pergigian tidak terkecuali daripada penggunaan gelatin dalam formulasinya. Kajian ini menggunakan spektroskopi inframerah transformasi total refleksi-Fourier yang dilemahkan (ATR-FTIR) ditambah dengan analisis komponen utama (PCA) untuk mengesan dan membezakan antara gelatin babi dan lembu dalam bahan pergigian. Hasilnya disahkan lebih lanjut dengan ujian reaksi berantai polimerase (PCR). Analisis ATR-FTIR dilakukan untuk menghasilkan data spektral untuk 42 sampel bahan pergigian. Spektrum dibandingkan dengan spektrum standard gelatin lembu dan babi. Semua data yang diperoleh dari ATR-FTIR dikenakan pra-pemprosesan data diikuti oleh PCA. PCR dijalankan untuk mengesahkan DNA babi dan lembu pada bahan pergigian. Primer khusus spesies yang mensasarkan 212 bp gen *sitokrom b* babi dan 271 bp gen *sitokrom b* lembu digunakan untuk mengesan DNA dalam sembilan sampel bahan pergigian. Pengesanan dan pembezaan standard gelatin (lembu dan babi) terhadap gelatin yang terdapat pada bahan pergigian dicapai dengan menggunakan ATR-FTIR yang digabungkan dengan PCA dalam bilangan gelombang 1756 cm^−1^–1584 cm^−1^ (Amide I dan Amide II). Had pengesanan untuk DNA masing-masing 0.001 ng/μL dan 0.0001 ng/μL untuk gelatin bovine dan porcine. Dengan menggunakan PCR, satu sampel, BDM 01, didapati mengandungi DNA babi dan lembu, sementara satu sampel (BDM 14) didapati positif DNA lembu. Hasil kajian menunjukkan bahawa ATR-FTIR yang digabungkan dengan PCA dan PCR konvensional dapat digunakan untuk mengenal pasti gelatin babi dan sapi dalam bahan pergigian.

**Highlights**

- Present study focuses on the detection of porcine and bovine gelatin in dental materials using different analytical methods.
- Analysis of dental material spectra via PCA was carried out only within the wavelength of 1756 cm^−1^ to 1584 cm^−1^, which correspond to the Amide I and Amide II spectral regions.
- Verification of porcine and bovine DNA in dental materials was successfully performed using conventional PCR.

## INTRODUCTION

Gelatin can be found not only in pharmaceutical products such as capsules but also in dental materials. The animal origin of gelatin used in products has been a source of concern amongst consumers for many reasons. It is reported that most commercial gelatins are produced from animals such as cattle and pigs. Gelatin sourced from pigskin is significantly more abundant than from other animals (GMIA 2012). Other animal sources of gelatin include chicken and fish (Liu *et al*. 2015). Gelatin has a wide range of applications for gelling, binding, foaming, emulsifying, thickening, moisture retaining, plasticising and texture improving of various products (Karim & Bhat 2008).

The authentication of gelatin sources is vital for both religious and safety concerns. Porcine-based products cannot be consumed by Muslims and Jews, while cow-based products cannot be eaten by Hindus because of their sacred position in religion. Bovine ingredients sourced from cattle infected with bovine spongiform encephalopathy (BSE) were previously a source of major consumer concern (Kim *et al*. 2013). In dental products, labeling of ingredients remains poor; many products disclose only the active ingredients, whereas they also contain inactive ingredients called excipients which improve the physical qualities of the product, such as colouring agents, thickening agents, diluents, and flavourings that may also include gelatin (Aziz *et al*. 2014). Detection of gelatin is essential for religious and safety reasons, as most materials applied during dental treatment come into contact with the patient’s saliva and blood, especially during tooth extraction. This contact may be considered a point of consumption or contamination with the material source, which may be against the patients’ religious requirements. Knowledge of the source of ingredients in dental materials could protect the consumer’s religious beliefs, especially Muslim consumers. Apart from religious reasons, the labeling of porcine or bovine gelatin in dental materials is crucial for patients with gelatin allergies (Doi *et al*. 2009).

Dental materials can be used in preventive, restorative, or auxiliary procedures, and have been classified under these categories. Preventive dental materials protect the teeth from dental caries, and normally consist of fluoride and other therapeutic agents in its formulation. Meanwhile, restorative dental materials enable reparation and replacement of the tooth structure, and are made of synthetic material (Powers & Wataha 2013). Meanwhile, auxiliary dental materials such as impression materials are used to fabricate dental prostheses (Anusavice *et al*. 2013). In Malaysia, dental materials are usually supplied from foreign countries and may include gelatin in their formulation. A few examples of dental materials that may contain gelatin are dental hemostatic agents, dental prophylaxis pastes, and toothpaste. The exact composition of these materials is unclear because their labels do not list down all the ingredients used in the formulation and fabrication, which could include gelatin. Muslim patients need to seek halal (permissible) dental materials. Meanwhile, dental practitioners need to ensure that they treat their patients with halal products as part of their professional integrity and religious responsibility.

ATR-FTIR is successfully able to detect raw gelatin in its pure form, but not in mixture (Hashim *et al*. 2010). Previous reports on authentication methods focused on the identification of the species of meat; in contrast, reports on gelatin identification are few. Analytical methods used in the detection of gelatin source include amino acid (Raja Mohd Hafidz *et al*. 2011; Raraswati *et al*. 2014), spectroscopic (Abdul Aziz *et al*. 2017; Cebi *et al*. 2016; Hashim *et al*. 2010; Hermanto *et al*. 2013) and DNA analysis (Cai *et al*. 2012; Demirhan *et al*. 2012; Lee *et al*. 2016; Mohamad *et al*. 2016; Muñoz-Colmenero *et al*. 2016; Nikzad *et al*. 2017; Sahilah *et al*. 2012; 2015; Shabani *et al*. 2015; Sudjadi *et al*. 2015; Tasara *et al*. 2005). Detection of gelatin source based on amino acid composition and protein is successfully able to differentiate gelatin origin in pure form but not in gelatin mixture, food and pharmaceuticals (Raraswati *et al*. 2014). This is due to protein degradation caused by extreme pH and temperature during the production of food and pharmaceuticals. Enzyme-linked immunosorbent assay has also been utilised to distinguish gelatin sources but less sensitive and less species-specific (Hashim *et al*. 2010).

Studies on the detection of porcine gelatin in dental materials are limited. A previous study reported the authentication of halal dental materials; however, the study was specific to analysis of orthodontic dental materials using ATR-FTIR (Abdul Aziz *et al*. 2017). Here, we report the detection of bovine and porcine gelatin in other types of dental materials was carried out using ATR-FTIR combined with PCA and further verified by PCR. PCR as a verification method for confirming the presence of bovine or porcine gelatin is advantageous; compared to protein, DNA is able to withstand high temperature and pressure treatments (Soares *et al*. 2013). Additionally, authentication of porcine and bovine gelatin via PCR has shown greater success due its higher specificity and sensitivity compared to ATR-FTIR (Cai *et al*. 2012; Lee *et al*. 2016; Nikzad *et al*. 2017; Shabani *et al*. 2015).

## MATERIALS AND METHODS

### Sample Preparation

Porcine and bovine skin gelatin powders were procured from Sigma-Aldrich (St. Louis, MO, USA) (Table 1). Gelatin derived from the skin was selected as standard over bone gelatin since the gelatin yield is highest in pig skin, which represents 46% of the total gelatin production (Duconseille *et al*. 2015). Forty-two dental material samples were procured from Malaysian dental suppliers. The dental materials were in powder, paste, gel and liquid forms. All the dental materials were placed onto the ATR surface area except for powder samples, which were homogenised and dissolved in deionised water before analysis. Deionised water was added to the pure porcine and bovine gelatin powders used as control standards, and the solution was heated for 10 min at 50°C to allow complete dissolution of the gelatin. The gelatin was allowed to cool to room temperature (15°C–25°C). The gelatin jellies were then placed on the ATR surface area for analysis (Hashim *et al*. 2010).

### Infrared Spectroscopy Measurements

The spectra were measured at a wavelength range of 4000-400 cm^−1^ with a 2 cm^−1^ resolution at ambient temperature (25 ± 0.5°C) using an ATR-FTIR spectrometer (PerkinElmer, Inc., USA). For each sample, 16 scans were performed, and two replicate spectra were obtained. The background air spectrum was subtracted from the spectral data. Before sample placement for analysis, the ATR element (ZnSe crystal, 45° ends) was cleaned with acetone and the results were recorded in absorbance units.

### Data Pre-Processing

Data pre-processing was performed to optimise spectral data before PCA. Each spectrum was baseline corrected, smoothed, normalised and subjected to the second derivative.

### Principal Component Analysis (PCA)

PCA was performed using Unscrambler^®^ X version 10.4 (Camo, USA) to classify and discriminate the samples. PCA algorithms were used to reduce the high-dimensional spectroscopic dataset by computing a linear combination of the original variable into a few orthogonal principal components. Spectra of gelatin standards, dental material samples (BDM 01, BDM 02, BDM 03, BDM 05, BDM 14, BDM 16, BDM28, BDM 31, and BDM 36) and porcine and bovine dental paste were analysed within the 1756 cm^−1^–1584 cm^−1^ wavelength. The total of 172 wavenumber values within 1756 cm^−1^–1584 cm^−1^ were set as variables, whereas the absorbance value for each sample was used as input data.

### Polymerase Chain Reaction (PCR)

#### DNA extraction

DNA was extracted from bovine and porcine gelatins (Table 1) and from nine samples of dental material containing gelatin (BDM 01, BDM 02, BDM 03, BDM 05, BDM 14, BDM 16, BDM28, BDM 31, and BDM 36) using DNeasy *Mericon* Food Kit (Qiagen, Germany). The small fragment protocol (200 mg; recommended for highly processed foods or pharmaceuticals where DNA has been subjected to extensive thermal treatments, irradiation, and pH changes or drying) outlined by the manufacturer was followed.

Briefly, 1000 μL of lysis buffer and 2.5 μL Proteinase K solution were added to 200 mg of sample and mixed by vortexing. The mixture was then incubated in a water bath for 30 min with constant shaking (1000 rpm) and cooled to room temperature (15°C–25°C) on ice. After cooling, the mixture was centrifuged for 5 min at 2500 x g. 700 μL of the clear supernatant was transferred to a new microcentrifuge tube containing 500 μL of chloroform and mixed by vortexing for 15 s followed by centrifugation at 14 000 x g for 15 min. Subsequently, 250 μL of the upper aqueous phase was added to a new 2 mL microcentrifuge tube containing 1000 μL buffer PB and mixed thoroughly by vortexing. This step was repeated twice to achieve higher DNA yields. 600 μL of the mixture was transferred into a spin column placed in a 2 mL collection tube, followed by centrifugation at 17 900 x g for 2 min, and the flow-through was discarded. This step was repeated twice. Next, 500 μL of wash buffer (Buffer AW2) was added to the spin column, centrifuged at 17 900 x g for 2 min and the flow-through was discarded. The collection tube was centrifuged again at 17 900 x g for 4 min to dry the membrane. After centrifugation, the spin column was transferred to a new 2 mL microcentrifuge tube, and 30 μL of elution buffer (Buffer EB) was added directly into the spin column membrane and incubated at room temperature (15°C–25°C) for 1 min. Finally, the spin column was centrifuged at 17 900 x g for 2 min to elute the DNA. The extracted DNA was stored at −20°C until use.

#### DNA quantification and purity

The extracted DNA was analysed for concentration and quality (purity) using a Thermo Fisher Scientific Nanodrop 1000 spectrophotometer (Thermo Fisher, USA). Prior to analysis, the lower measurement pedestal was cleaned with lab wipes (Kim wipes™). Elution buffer was used as a blank before analysis. Next, 2 μL of DNA sample was pipetted onto the lower measurement pedestal. The spectral measurement was then initiated. The concentration of DNA from each sample was quantified by measuring optical density (OD) at 260 nm wavelength. Meanwhile, purity of the DNA sample was determined by measuring OD at 260/280 nm.

### DNA amplification by PCR

#### Species-specific primers and PCR amplification

Species-specific primers for bovine and porcine DNA were obtained from First Base Laboratories (Selangor, Malaysia). Information on the bovine and porcine primers is listed in Table 2. The species-specific primers were designed to target mitochondrial DNA (mtDNA) of the cytochrome *b* gene.

Amplification of DNA was carried out via conventional PCR. Each reaction was performed in a final volume of 25 μL containing 12.5 μL of REDiant 2X master mix (First Base, MY), 10 ng of DNA template, 1 μL each of 10 μM forward and reverse primers, and nuclease-free water. Negative and positive controls were prepared by adding nuclease-free water and porcine or bovine DNA to the tubes, respectively.

PCR amplification was performed in a T100 thermal cycler (Bio-Rad, Hercules, USA) under the following conditions: initial denaturation for 4 min at 94°C, followed by 34 denaturation cycles for 30 s at 94°C, annealing for 40 s at 58°C and extension for 30 s at 72°C. Then, a final extension was performed for 5 min at 72°C.

### Gel Electrophoresis

#### Electrophoresis and gel documentation

The amplified PCR products were analysed using 1.7% agarose gel in 1X Tris-acetate-EDTA (TAE) buffer with fluorosafe DNA stain (First Base, Malaysia) as a visualising agent. First, the prepared agarose gel was placed inside the electrophoresis chamber, followed by adding enough 1X TAE buffer to cover the gel. Five μL (5 μL) of DNA ladder, 8 μL of PCR product, and negative control were loaded into the wells. Electrophoresis was run at 100 V for 45 min using a Cleaver Scientific electrophoresis system. A 100 bp DNA ladder (Genedirex, USA) was used as a size marker, and the amplified DNA was visualised using a Chemidoc XRS^+^ imaging system (Bio-Rad, USA).

### Specificity and sensitivity of conventional PCR assays

Specificity of the porcine and bovine primers was evaluated in DNA extracted from originally sourced porcine and bovine skin gelatin powder. The detection limit of the assay was determined by analysing serial dilutions of DNA extracted from the porcine and bovine skin gelatins with DNA concentrations starting at 10 ng/μL.

## RESULTS

### ATR-FTIR Detection of Porcine and Bovine Gelatin in Dental Material

#### Analysed dental materials classification

Table 3 lists all the 42 dental material samples (BDM01 – 42) used in this study, categorised based on the material type and batch number. Material types were classified as hemostatic agents, restoration materials, dental prophylactic agents, oral surgery materials, impression material, dental anesthetic agents, oral rinses, prosthetic materials and preventive materials. The inclusion of gelatin in the list of ingredients as well as country of production was also recorded. Nine out of 42 samples were found to contain gelatin from either porcine or bovine sources. Four of the samples were categorised under haemostatic agents, two under restorative materials and one sample each under dental prophylaxis, impression material and oral rinse.

### Spectra of dental materials and control samples

**Figure 1:**
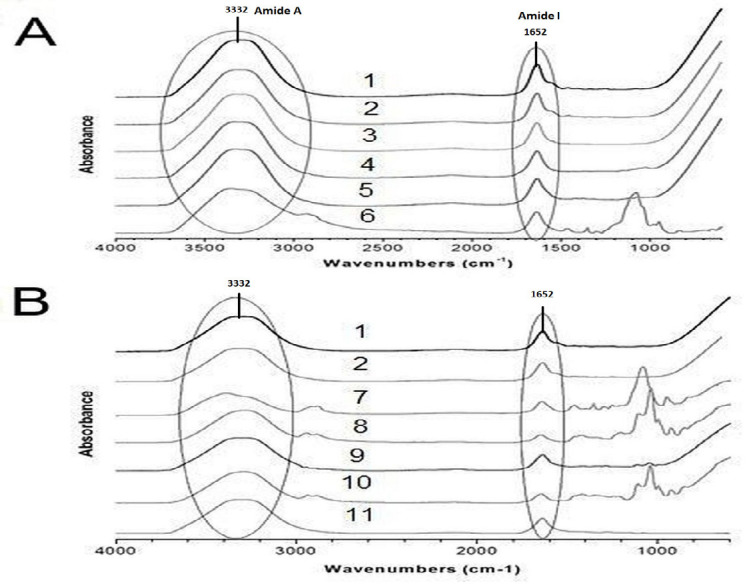
(A) Hemostatic agents ATR spectra (1: bovine gelatin; 2: porcine gelatin; 3: BDM 01; 2: BDM 02; 4: BDM 03; 5: BDM 05) and (B) other dental material samples ATR spectra (7: BDM 14; 8: BDM 16; 9: BDM 28; 10: BDM 31; 11: BDM 36) showed similar peaks with the controls’ ATR spectra, marked with circles

The ATR-FTIR spectra of nine dental materials containing gelatin compared against control samples (porcine and bovine gelatin) are shown in Fig. 1. Both porcine and bovine gelatin showed similar spectral patterns at four regions: 3600–2300 cm^−1^ (Amide A), 1656–1644 cm^−1^ (Amide I), 1560–1335 cm^−1^ (Amide II), and 1240–670 cm^−1^ (Amide III). All nine samples showed similar patterns to the control in the 3700–3100 cm^−1^ and 1700–1600 cm^−1^ regions corresponding to the Amide A and Amide I regions, respectively.

### Principal component analysis

FTIR spectral data is multivariate, which means that the data includes hundreds of wavenumbers. Every datum point in the spectrum is profoundly affected by subsequent data points due to overlaps in the biomolecule absorbance, especially in protein samples (Allison 2011). Since the spectra of protein samples are strongly influenced by the covariance between biological constituents, it is difficult to differentiate one protein spectrum from another, especially when the spectra structures are identical. One of the methods for the analysis of multivariate data is using PCA. PCA reduces the number of variables in a multidimensional dataset while retaining variation within the datasets (Varmuza 1998). The use of ATR-FTIR alone is insufficient to discriminate between different species of gelatin due to high spectral structure similarities between the control and sample data. Hence, PCA was used to differentiate between spectra of controls, dental materials and dental paste samples comprising bovine and porcine gelatins.

**Figure 2:**
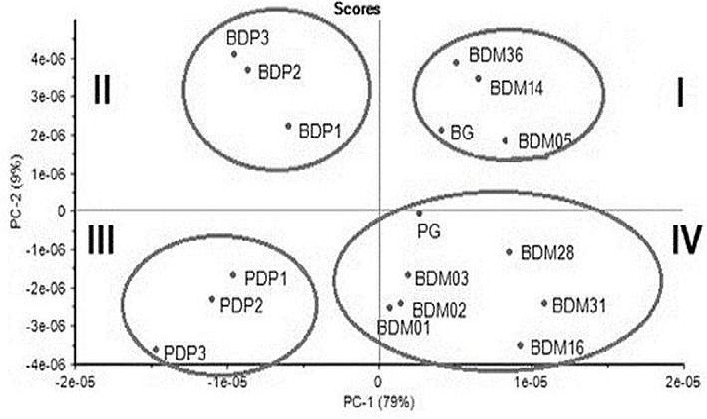
Score plot expressed as first principal component (PC1) and second principal component (PC2) for the classification of gelatin standards (PG: porcine gelatin; BG: bovine gelatin), experimental gelatin-containing dental paste (PDP: porcine gelatin-containing dental paste; BDP: bovine gelatin-containing dental paste and unknown samples of dental material (BDM01; BDM02; BDM03; BDM05; BDM14; BDM16; BDM31; BDM36). I, II, III, and IV refer to quadrants I, II, III and IV.

In order to evaluate possible classes among bovine and porcine gelatin standards, toothpaste samples containing porcine and bovine gelatin as well as dental material samples containing gelatin of unknown sources were analysed using ATR–FTIR within the 1756 cm^−1^–1584 cm^−1^ wavelength region. Each spectrum was corrected for baseline, smoothed, area normalised, and subjected to the second derivative. The results are presented in Fig. 2.

Based on Fig. 2, two principal components were identified: the first principal component (PC1) and second principal component (PC2). PC1 and PC2 described 79% and 9% of the data variability. Thus, the total variation in the spectral data (PC1 and PC2) was 88%. Based on the score plot, the bovine and porcine gelatin standards (control samples) were separated into two different quadrants which are quadrants I and IV. Experimental bovine- and porcine-containing gelatin toothpaste could also be distinguished according to species as the samples were clustered in different quadrants, namely quadrants II and III. Unknown samples such as BDM 36, BDM 14 and BDM 05 were clustered together with the bovine gelatin standard and showed positive PC2 score values.

**Figure 3:**
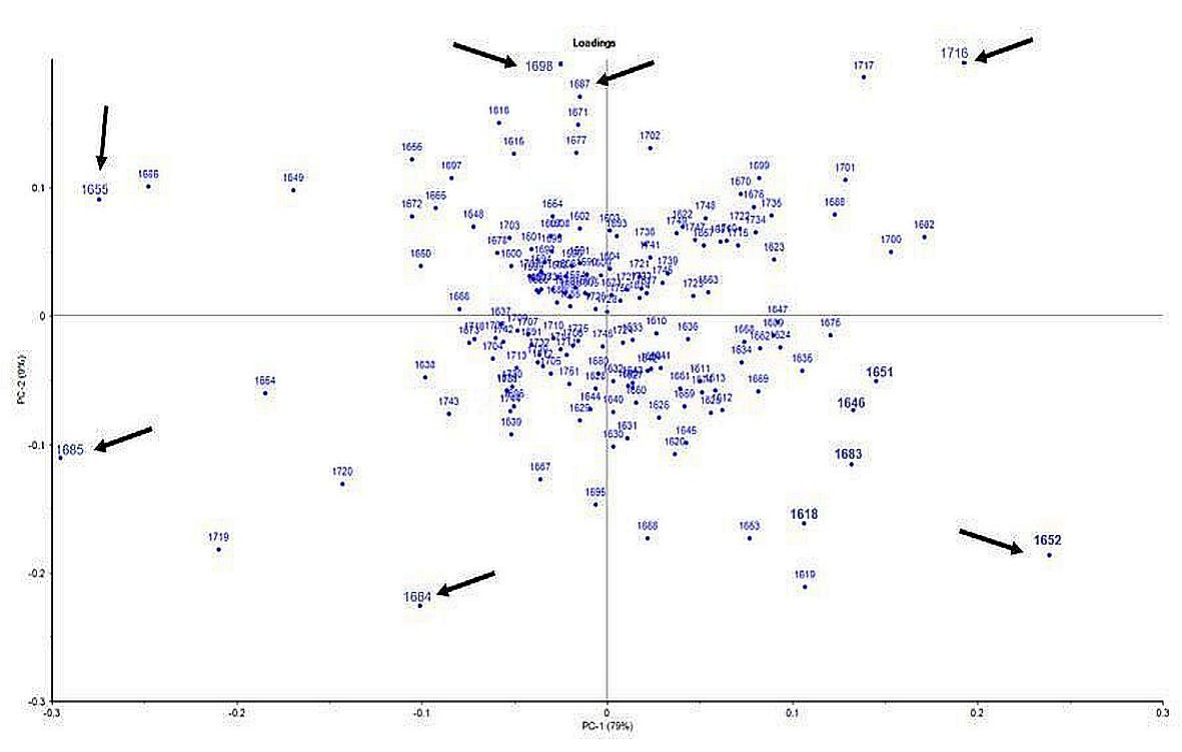
Loading plot for the classification of porcine and bovine gelatins in dental materials. The wavenumbers that strongly contributed to the variable separation for PC1 and PC2, respectively were shown with arrows.

Meanwhile, BDM01, BDM02, BDM03, BDM 16, BDM 31, and BDM 28 were grouped with porcine gelatin, which showed negative PC2 score values. The loading plot (Fig. 3) displays how each variable contributes to the loading of each component. The separation of these classes could be determined from the loading plot, which highlights the most critical features of the dataset, such as wavenumbers. Variables located farther from the origin shows a higher contribution to the PCA model (Marina *et al*. 2010). The wavenumbers that strongly contribute to the variable separation for PC1 are shown on the far right (1652 cm^−1^ and 1716 cm^−1^) and the far left (1655 cm^−1^ and 1685 cm^−1^). Based on PC2, the separation of classes was contributed by wavenumbers 1684 cm^−1^, 1687 cm^−1^ and 1698 cm^−1^. This study also reports that only one sample, BDM 01, was consistent with labeling information provided by the supplier stating that the product was derived from porcine gelatin.

### PCR Detection of Porcine and Bovine Gelatin in Dental Materials

#### DNA extraction quantitative analysis

In gelatin, DNA is more stable than proteins (Linacero *et al*. 2016). Hence, DNA analysis was performed to further verify the presence of bovine and porcine sources in the nine dental material samples following ATR-FTIR. PCR amplification requires sufficient DNA template for analysis. In highly processed foods or pharmaceuticals, DNA is degraded into short fragments that may cause difficulties in PCR amplification. Hence, it is crucial to obtain sufficient DNA for use as a template before analysis using PCR amplification. DNA extraction was carried out using a commercial kit under optimised column-binding conditions adjusted to recover sufficient amounts of short DNA fragments. The quantity and purity of DNA templates extracted from 200 mg of standard bovine and porcine gelatin powders and nine dental material samples previously analysed by ATR-FTIR are presented in Table 4. The concentration of extracted DNA from porcine and bovine gelatin and dental material samples ranged from 2.90 ng/μL to 20.04 ng/μL. The purity of the DNA template ranged from 1.61 to 1.87. Although the extracted DNA from some samples was low, the overall DNA purity (A260/A280) was high, indicating that high-quality DNA was successfully extracted.

### Specificity and sensitivity of PCR assay

**Figure 4:**
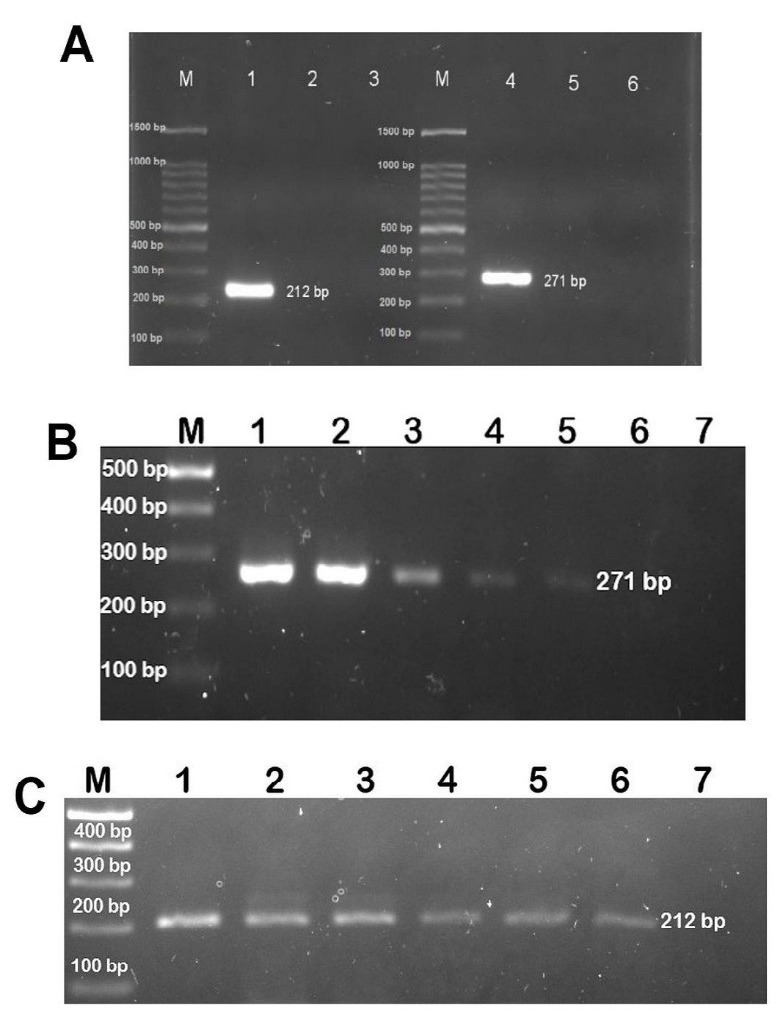
(A) Specificity of porcine and bovine-specific primers. Lanes 1 and 2 are porcine and bovine DNA, respectively tested with porcine primers; lanes 4 and 5 are porcine and bovine DNA tested with bovine primers. Lanes 3 and 6 are no template control (NTC); M: DNA marker (100 bp ladder). Detection limit of PCR assay using specific primers for bovine (B) and porcine (C). M: DNA marker (100 bp ladder), lane 1: 10 ng/μL, lane 2: 1 ng/μL, lane 3: 0.1 ng/μL, lane 4: 0.01 ng/μL, lane 5: 0.001 ng/μL, lane 6: 0.0001 ng/μL and lane 7: no template control (NTC).

Small fragment amplification of mitochondrial genes is recommended for the analysis of DNA extracted from highly processed products. Hence, the present study utilised species-specific primers targeting the mtDNA cytochrome b (*Cyt b*), which was previously reported in bovine and porcine DNA detection studies in various products. To evaluate primer specificity, both primers were used to amplify DNA extracted from gelatin powders of known bovine and porcine sources. As shown in Fig. 4A, gel electrophoresis carried out on the amplified PCR products from porcine and bovine gelatin produced expected bands of 212 and 271 base pairs (bp), respectively. No cross-reaction was observed.

Next, the sensitivity of the assay was determined by analysing serial dilutions of the DNA extracted from porcine and bovine skin gelatins with DNA concentrations starting at 10 ng/μL. The DNA template extracted from bovine and porcine gelatin powders was diluted in 10 times serial dilution, corresponding to a DNA concentration ranging from 1 ng/μL to 0.0001 ng/μL. As shown in Fig. 4B, the detection limit for bovine DNA was 0.001 ng/μL, while porcine DNA (Fig. 4C) was detectable in amounts as low as 0.0001 ng/μL. These results indicate that conventional PCR is sensitive enough to detect the presence of very low amounts of bovine and porcine gelatins.

### Application of species-specific PCR assays to dental material samples

**Figure 5:**
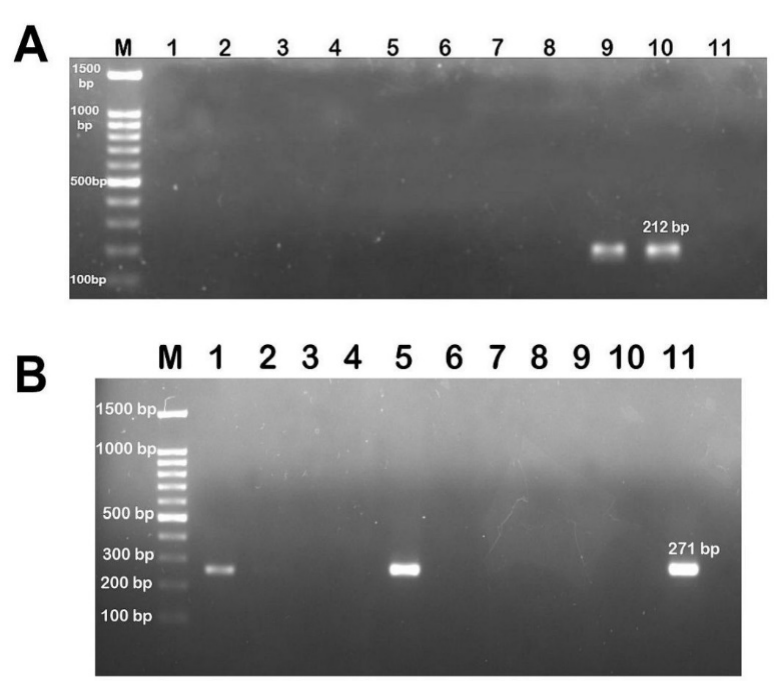
Agarose gel electrophoresis of PCR products amplified from extracted DNA of dental materials tested with porcine-specific primer pairs (A). Lanes 1–9: dental material samples, lane 10: positive control (212 bp), lane 11: no template control (NTC) and M: DNA marker (100 bp ladder). Agarose gel electrophoresis of PCR products amplified from extracted DNA of dental materials tested with bovine-specific primer pairs (B). Lanes 1–9: dental material samples, lane 10: no template control (NTC) and lane 11: positive control (271 bp) and M: DNA marker (100 bp ladder).

Next, PCR was used to verify the presence of bovine and porcine DNA in dental material samples. Nine dental material samples were subjected to DNA extraction. The isolated DNA was amplified in a thermal cycler using the bovine and porcine primers following optimised PCR conditions to determine the gelatin species present in the samples. One sample (BDM 01) was found to contain porcine gelatin, while the remaining eight samples (BDM 02, 03, 05, 14, 16, 28, 31 and 36) were negative for porcine gelatin (Fig. 5A). The findings were consistent with the labeling information provided by the supplier on sample BDM 01, which was derived from pure porcine gelatin.

Meanwhile, two samples (BDM 01 and 14) were found to be positive for bovine gelatin, revealing PCR amplification products 271 bp in size, while the remaining samples were found to be negative for bovine gelatin (Fig. 5B). The findings in these two samples were inconsistent with labeling information, as the label did not list bovine-derived materials. Interestingly, BDM 01 was found to contain both bovine and porcine DNA, although the label only listed porcine gelatin in its list of ingredients.

## DISCUSSION

Most commercial gelatin is derived from the hides and bones of mammals, such as cows and pigs (Shabani *et al*. 2015). Additionally, gelatin can also be derived from fish (Karim & Bhat 2009). Owing to various factors such as availability, religion concerns, cost, region, and others, gelatin can be produced from different sources. According to Venugopal *et al*. (2008) porcine gelatin is the most widely used type of gelatin because it possesses higher gel strength, is stress-resistant, can hold water, and has economic value, unlike other sources such as bovine and fish gelatin. Muslims, Jews, Hindus, vegans and vegetarian communities place great concern on the source of gelatin from which they consume (Boran & Regenstein 2010). For this reason, gelatin authentication is essential to safeguard the consumer from non-*halal* ingredients as well as to protect their health and safety. It has been reported that the use of bovine-based products may be associated with BSE, a fatal neurodegenerative disease in cattle that can lead to brain and spinal cord degeneration (Widyaninggar *et al*. 2012).

The use of gelatin in products primarily in the pharmaceutical and medical industries has been widely reported (Freedman & Stassen 2013; Sahilah *et al*. 2012; Sileshi *et al*. 2008). Gelatin is added to these products because of its unique properties in the physiologic environment; gelatin is enzyme biodegradable and biocompatible. In the present study, gelatin detection in dental material samples was verified through PCR. The *halal* status of dental materials is generally given less attention, especially when compared to food products. There is no *halal* requirement for most dental products, and as they are imported from foreign countries, the ingredients used in their fabrication could be from doubtful sources. In dentistry, some dental products may be made from non-*halal* sources, such as porcine sources (Halib *et al*. 2016). Therefore, there is a need for reliable, rapid, and sensitive methods to detect the presence of nonhalal substances in dental products.

The present study evaluates the use of ATR-FTIR to compare the spectra of gelatin standards to the spectrum of each dental material sample. Our results show that porcine and bovine gelatin spectra showed similar patterns in the following four regions: 3600 cm^−1^–2300 cm^−1^ (Amide A), 1656 cm^−1^–1644 cm^−1^ (Amide I), 1560 cm^−1^–1335 cm^−1^ (Amide II), and 1240 cm^−1^–670 cm^−1^ (Amide III). These results were in agreement with studies by Cebi *et al*. (2016), Hashim *et al*. (2010) and Muyonga *et al*. (2004) where similar peaks were observed. Due to the denaturation of collagen in gelatin, the gelatin spectrum showed low intensities of the Amide I and Amide II bands. Meanwhile, the Amide III band was almost non-existent. The poor intensity of the Amide III band could be due to the loss of triple helix molecules during high temperature extraction of gelatin (Krimm & Bandekar 1986).

Amide I absorption denotes carbonyl C=O (peptide bond) stretching, with less contribution of the C-N bond stretching. Amide II absorption is due to bending of the N-H bond and stretching vibration of the C-N bond (Krimm & Bandekar 1986). All three regions namely, Amide I, II and III have been reported to be beneficial in the detection of protein secondary structures (Jackson & Mantsch 1995; Srivastava *et al*. 2011). The C-N stretching vibrations coupled with N-H in-plane bending vibrations, weak contributions from C-C bond stretching vibrations and C=O in-plane bending give rise to Amide III absorption (Bandekar 1992). Thus, Amide III shows a less defined vibrational mode with varying protein vibrations (Bandekar 1992).

Most samples with peaks similar to the control spectra were hemostatic agents. Hemostatic agents control bleeding during tooth extraction, oral surgery, and implants (Mani *et al*. 2014). Dental hemostatic agents were reported previously to contain bovine and porcine gelatins (Schreiber & Neveleff 2011). These types of haemostatic agents are called mechanical haemostats which act as first-line agents in the control of minor bleeding (Corwin *et al*. 2004). Besides, collagen derived from bovine sources were previously reported to be effective in controlling hemorrhage (Spotnitz & Burks 2010).

ATR-FTIR combined with PCA could be applied as a method to screen, identify, and discriminate between porcine and bovine gelatins present in commercial products. Successful identification of gelatin from different species was previously reported using FTIR combined with chemometric analysis (Cebi *et al*. 2016; Hashim *et al*. 2010). Porcine and bovine gelatin discrimination was reported using ATR-FTIR combined with PCA at the 1756 cm^−1^ to 1584 cm^−1^ wavelengths, which correspond to the Amide I and Amide II spectral regions. Previous studies by Cebi *et al*. (2016) report that discrimination and classification of pure bovine and porcine gelatin samples could be accomplished using the Amide I and Amide II spectral regions specifically, where bands were observed at around 1722 cm^−1^–1487 cm^−1^. However, a study by Hashim *et al*. (2010) reported that the discrimination of gelatin was successfully achieved using spectral ranges of 3290 cm^−1^–3280 cm^−1^ and 1660 cm^−1^–1200 cm^−1^. The Amide I region has been used widely in protein studies because of the presence of strong band frequencies that are associated with the protein secondary structure (Bestley 2007). This study also reports that only one sample, BDM 01, was consistent with labeling information provided by the supplier stating that the product was derived from porcine gelatin.

Generally, DNA extracted from gelatin is highly degraded due to the various processing steps involved in gelatin production. Mitochondrial genes such as those coding for 12S rRNA, 16S rRNA, 18S rRNA, *Cyt b*, cytochrome oxidase II, and NAD dehydrogenase, are commonly utilised for the detection of specific animal species in gelatin. Identification of mtDNA is reliable since mtDNA genes are present in thousands of copies per cell. Besides, mtDNA (circular) genes are more stable and exist intracellularly compared to nuclear (linear) DNA (Montiel-Sosa *et al*. 2000). Small fragment amplification of mitochondrial genes is suggested for the analysis of DNA extracted from highly processed products. Hence, the present study utilised species-specific primers targeting the mtDNA, *Cyt b*, which was previously utilised in bovine and porcine DNA detection studies in various products. The primers targeting *Cyt b* were able to detect porcine and bovine gelatin in the dental materials. The findings were in agreement with previous studies reported by Amin *et al*. (2017) where mitochondrial genes encoding the 100 bp porcine *Cyt b* gene were targeted to quantify porcine DNA in formulated feeds. Mitochondrial DNA was chosen because of its higher detection sensitivity compared to single or low-copy nuclear DNA targets.

Based on our findings using conventional PCR, previously published species-specific porcine and bovine primers can be used to detect porcine and bovine DNA in dental materials (Lahiff *et al*. 2001; Shabani *et al*. 2015; Tartaglia *et al*. 1998; Tasara *et al*. 2005). The reported species-specific primers were previously used to detect porcine and bovine DNA in various products including pharmaceuticals, animal feed, and meat. Shabani *et al*. (2015) employed the same porcine and bovine primers to authenticate gelatin in pharmaceutical capsule shells collected from pharmacies in Tehran. The primers used were capable of detecting bovine and porcine DNA in these products.

The primers used in this study were found to be sensitive to DNA concentrations of as low as 0.001 ng/μL and 0.0001 ng/μL for bovine and porcine gelatins, respectively. Findings from previous studies also report that PCR is able to detect porcine and bovine DNA in materials that have undergone extensive physical and chemical changes (Cai *et al*. 2012; Lee *et al*. 2016; Malik *et al*. 2016; Nikzad *et al*. 2017; Sahilah *et al*. 2012). The DNA found in these products is generally present in low amounts and in highly degraded form due to the destructive methods during gelatin production and processing. Lee *et al*. (2016) reported detection limits of bovine and porcine primers at 0.001 and 0.01 ng/μL, respectively.

## CONCLUSION

The present study focuses on the detection of porcine and bovine gelatin in dental materials using different analytical methods. A combination of ATR-FTIR and PCA analysis was able to detect gelatin species in the dental material samples, as the use of PCA showed 88% of the data variation. Analysis of dental material spectra via PCA was carried out only within the wavelength of 1756 cm^−1^ to 1584 cm^−1^, which correspond to the Amide I and Amide II spectral regions. These spectral regions allowed for better distinction between bovine and porcine gelatin in dental material samples. Verification of porcine and bovine DNA in dental materials was successfully performed using conventional PCR with sensitivity levels of up to 0.001 ng/μL and 0.0001 ng/μL for bovine and porcine DNA, respectively. Therefore, ATR-FTIR combined with PCA and conventional PCR are useful for identifying bovine and porcine gelatin in highly processed dental materials. These analytical methods could be further applied in routine investigations for halal authentication of foods and pharmaceutical products containing gelatin to protect consumers from non-halal product consumption.

## Figures and Tables

**Table 1:** General description of the gelatin standards used in this study.

Gelatin source	Company name	Gelatin type	Bloom value
Gelatin from bovine skin	Sigma Aldrich	Type B	~225
Gelatin from porcine skin	Sigma Aldrich	Type A	~300

**Table 2:** Species-specific primers used in this study.

Species	Primer sequences 5′to 3′	Amplicon	Target gene	Annealing temperature	Reference
Bovine	5′GCCATATACTCTCCTTGGTGACA3′	271 bp	*Cyt b*	58°C	(Tartaglia *et al*.1998)
*Bos taurus*	5′GTAGGCTTGGGAATAGTACGA3′				(Shabani *et al*. 2015)
Porcine	5′GCCTAAATCTCCCCTCAATGGTA3′	212 bp	*Cyt b*	58ºC	(Lahiff *et al*. 2001)
*Sus scrofa*	5′ATGAAAGAGGCAAATAGATTTTCG3′				(Shabani *et al*. 2015)

**Table 3:** List of available dental material samples and gelatin presence in dental material samples using ATR-FTIR spectroscopy.

Number	Material description	Material type	Gelatin label declaration	Manufacturing country	Gelatin presence
BDM 01	Hemostatic agent	Sponge	Yes	USA	Yes
BDM 02	Hemostatic agent	Sponge	No	USA	Yes
BDM 03	Hemostatic agent	Sponge	No	USA	Yes
BDM 04	Hemostatic agent	Liquid	No	Switzerland	No
BDM 05	Hemostatic agent	Paste	No	USA	Yes
BDM 06	Restoration material	Liquid	No	Germany	No
BDM 07	Restoration material	Liquid	No	UK	No
BDM 08	Restoration material	Powder	No	France	No
BDM 09	Restoration material	Paste	No	USA	No
BDM 10	Restoration material	Powder	No	USA	No
BDM 11	Dental prophylaxis	Gel	No	USA	No
BDM 12	Restoration material	Paste	No	Germany	No
BDM 13	Restoration material	Liquid	No	USA	No
BDM 14	Restoration material	Paste	No	Germany	Yes
BDM 15	Dental anesthetic agent	Liquid	No	France	No
BDM 16	Dental prophylaxis	Paste	No	Germany	Yes
BDM 17	Restoration material	Paste	No	Germany	No
BDM 18	Oral surgery	Paste	No	Canada	No
BDM 19	Restoration material	Paste	No	USA	No
BDM 20	Impression material	Paste	No	Germany	No
BDM 21	Impression material	Gel	No	USA	No
BDM 22	Restoration material	Liquid	No	Germany	No
BDM 23	Restoration material	Paste	No	USA	No
BDM 24	Restoration material	Paste	No	USA	No
BDM 25	Impression material	Paste	No	USA	No
BDM 26	Restoration material	Paste	No	USA	No
BDM 27	Dental anesthetic agent	Gel	No	USA	No
BDM 28	Preventive material	Gel	No	USA	Yes
BDM 29	Dental anesthetic agent	Gel	No	USA	No
BDM 30	Preventive material	Liquid	No	USA	No
BDM 31	Oral rinse	Liquid	No	USA	Yes
BDM 32	Restoration material	Liquid	No	USA	No
BDM 33	Prosthetic material	Powder	No	USA	No
BDM 34	Periodontal dressing	Paste	No	USA	No
BDM 35	Restoration material	Paste	No	USA	No
BDM 36	Restoration material	Gel	No	USA	Yes
BDM 37	Dental prophylaxis	Paste	No	UK	No
BDM 38	Dental prophylaxis	Paste	No	Germany	No
BDM 39	Dental prophylaxis	Paste	No	USA	No
BDM 40	Dental prophylaxis	Paste	No	USA	No
BDM 41	Dental prophylaxis	Powder	No	USA	No
BDM 42	Dental prophylaxis	Paste	No	UK	No

**Table 4:** Extracted DNA concentrations and purities. The mean DNA concentration and purity ± SD from two replicates.

Sample	Mean extracted DNA concentration (ng/μL)	Mean extracted DNA purity [260/280 (ratio)]
Bovine gelatin powder	20.5 ± 0.69	1.87 ± 0.01
Porcine gelatin powder	11.5 ± 1.17	1.84 ± 0.04
BDM 01	3.0 ± 0.14	1.71 ± 0.01
BDM 02	34.8 ± 0.99	1.75 ± 0.01
BDM 03	9.0 ± 0.42	1.72 ± 0.01
BDM 05	7.2 ± 0.42	1.73 ± 0.02
BDM 14	8.9 ± 0.21	1.75 ± 0.01
BDM 16	6.7 ± 0.69	1.71 ± 0.01
BDM 28	7.3 ± 0.15	1.75 ± 0.04
BDM 31	6.0 ± 0.40	1.74 ± 0.02
BDM 36	9.5 ± 0.13	1.74 ± 0.01
